# Effects of dipyrone on the digestive tract

**DOI:** 10.1590/1414-431X20188103

**Published:** 2019-01-10

**Authors:** E.F. Collares, L.E.A. Troncon

**Affiliations:** 1 Departamento de Pediatria, Faculdade de Ciências Médicas, Universidade Estadual de Campinas, Campinas, SP, Brasil; 2 Departamento de Clinica Médica, Faculdade de Medicina de Ribeirão Preto, Universidade de São Paulo, Ribeirão Preto, SP, Brasil

**Keywords:** Dipyrone, Gastric emptying, Gastric mucosa, Sphincter of Oddi, Intestinal Motility, Adrenergic receptors

## Abstract

Dipyrone (metamizole), acting through its main metabolites 4-methyl-amino-antipyrine and 4-amino-antipyrine, has established analgesic, antipyretic, and spasmolytic pharmacological effects, which are mediated by poorly known mechanisms. In rats, intravenously administered dipyrone delays gastric emptying (GE) of liquids with the participation of capsaicin-sensitive afferent fibers. This effect seems to be mediated by norepinephrine originating from the sympathetic nervous system but not from the superior celiac-mesenteric ganglion complex, which activates β2-adrenoceptors. In rats, in contrast to nonselective non-hormonal anti-inflammatory drugs, dipyrone protects the gastric mucosa attenuating the development of gastric ulcers induced by a number of agents. Clinically, it has been demonstrated that dipyrone is effective in the control of colic-like abdominal pain originating from the biliary and intestinal tracts. Since studies in humans and animals have demonstrated the presence of β_2_-adrenoceptors in biliary tract smooth muscle and β_2_-adrenoceptor activation has been shown to occur in dipyrone-induced delayed GE, it is likely that this kind of receptors may participate in the reduction of smooth muscle spasm of the sphincter of Oddi induced by dipyrone. There is no evidence that dipyrone may interfere with small bowel and colon motility, and the clinical results of its therapeutic use in intestinal colic appear to be due to its analgesic effect.

## Introduction

Antipyrine (phenazone), a compound with a relatively simple structure synthesized around 1880, has been used as an antipyretic and analgesic agent (1,2). This drug is metabolized by mitochondrial enzymes in the liver and generates as a main product 4-hydroxyantipyrine and its conjugates, which are eliminated in the urine (2).

Further modifications of the antipyrine molecule aiming at improving its analgesic and antipyretic properties gave origin to various pharmaceuticals, such as aminophenazone (aminopiryne or amidopyrine), propyphenazone, dipyrone (metamizole), and phenylbutazone (1).

More recently, other pyrazolone derivatives have been synthesized and have experimentally demonstrated effective analgesic, anti-inflammatory, antiplatelet, and anticonvulsant activities, among other effects (3).

Clinically, dipyrone shows powerful analgesic, antipyretic, and antispasmodic effects, whereas its anti-inflammatory activity is considered irrelevant (4).

Dipyrone, as the most popular pyrazolone derivative, is a drug extensively employed therapeutically in some countries, whereas its use has been banned or restricted in others due to its possible undesired and dangerous reactions, such as agranulocytosis (4). Dipyrone is generally regarded as a pro-drug that generates *in vivo* larger metabolites, such as 4-methyl-amino-antipyrine (MAA), 4-amino-antipyrine (AA), 4-acetyl-amino-antipyrine (AAA), and 4-formyl-amino-antipyrine (FAA) (1,4). Its pharmacological effects are attributed to the active metabolites MAA and AA (1,4,5). In humans and rats, non-enzymatic hydrolysis of dipyrone generates MAA, which is subsequently metabolized in the liver under the action of cytochrome P450 subfamilies (6,7).

In the present text, detailed information is provided regarding the mechanisms of dipyrone pharmacological actions, and the effects on gastrointestinal motility, gastric mucosa, and the spasmolytic property of this drug.

## Material and Methods

For the present review, we analyzed papers available in the PubMed database up to December 2017, using the following key words, separately or in combination: dipyrone (metamizole), mechanisms, gastrointestinal motility, gastric emptying, gastric mucosa, colic pain, sphincter of Oddi, sympathetic nervous system, adrenergic receptors, cyclooxygenases (COXs). Studies in which dipyrone effects were assessed in combination with other drugs (composite preparations) were not included.

## Mechanisms of action

Although dipyrone is known as a powerful analgesic and antipyretic agent, the identification of the mechanisms involved in these effects lags considerably behind the advances observed regarding other drugs with similar properties.

Recent work has suggested possible mechanisms involved in the triggering of experimental endotoxic (lipopolysaccharide, LPS) fever by the rapid peripheral production of prostaglandin E_2_ (PGE_2_) followed by maintenance of its synthesis (8). This generates a pyrogenic message that reaches the central nervous system (CNS) through mechanisms involving both neuronal pathways (afferent pathway of the vagus nerve) and humoral pathways (8). Evidence obtained in experimental models has shown that the dipyrone MAA metabolite inhibits both PGE_2_-dependent and -independent fever, whereas AA blocks PGE_2_-dependent fever (9,10).

Dipyrone is currently classified as a non-opioid analgesic, although for a long time it was incorrectly included in the group of non-steroidal anti-inflammatory drugs (NSAID) (11). The mechanisms involved in its analgesic effect are not clearly understood and controversial results have been often reported.

Inhibition of cyclooxygenase 3 (COX-3), a variant of COX-1, by analgesic/antipyretic drugs with low anti-inflammatory activity such as dipyrone (including its MAA metabolite) and antipyrine may possibly be the primary CNS mechanism for the reduction of pain (12).

In addition to COX-3 inhibition, both the opioidergic and cannabinoid systems may be involved in the analgesic effect of the drug, or rather of its active metabolites (11). Studies in mice suggest the participation of endogenous cannabinoid and vanilloid systems (5,13,14) with activation of cannabinoid receptor type 1 (CB1) and transient receptor potential vanilloid 1 (TRPV1) receptors, respectively, in the periaqueductal-gray rostral ventromedial medulla axis of the CNS (14). In this animal model, parenterally administered dipyrone and antipyrine, but not MAA, blocked the transient receptor potential ankyrin 1 (TRPA1) channel. This receptor is expressed by peripheral sensory neurons with cell bodies in the dorsal root ganglia, functioning as antagonists of this channel, which, when activated, have a hyperalgesic nociceptive effect (15). On the other hand, another study concluded that arachidonoyl metabolites, which depend on the action of the fatty acid amide hydrolase, and cannabinoid receptors do not participate in the pharmacological effects of this drug under non-inflammatory conditions (16). In addition, a study also conducted in mice suggested that, in acute pain tests, cannabinoid CB1 receptors do not participate in the antinociceptive effect of dipyrone administered intraperitoneally, although the participation of these receptors in other pain models (neuropathy, inflammation, etc.) or in different routes of administration of the drug was not ruled out (17).

The involvement of the descending spinal pathways in the analgesic effect of dipyrone is controversial. One study concluded that the descending spinal adrenergic (α_2_-adrenoceptors) and serotonergic (5-HT receptors) pathways do not participate in this effect (18). More recently, however, evidence has been reported that various subtypes of serotonergic receptors (5-HT_2a_, 5-HT_3_, and 5-HT_7_-serotonergic) and adrenergic subtypes (α_1_, α_2_, β-adrenergic receptors) mediate the antinociceptive effect of dipyrone (19).

The central analgesic effect of this drug has been contested in a rat model of inflammatory pain (intraplantar injection of λ-carrageenan), thus suggesting that its peripheral action is not opioid-dependent (20).

It has been found in rats that thirty minutes after local administration of dipyrone the anti-hyperalgesic effect is associated to the activation of neuronal CB1 receptors induced by the AA metabolite and MAA-induced cGMP activation, with opening of the neuronal K_ATP_ channel (21). In contrast, another study also conducted in rats concluded that cannabinoid receptors are not involved in the peripheral antinociception induced by dipyrone (22). However, in the latter study the antinociceptive effect was evaluated 5 min after local (paw) administration of dipyrone and 15 min after local application of the CB1 and CB2 cannabinoid receptor antagonists in a model of hyperalgesia with previous local application of PGE_2_. Because of the need to generate active metabolites at the site of dipyrone application (23), the short time between application and evaluation of the effect in this study raises the possibility that the required local concentrations of these metabolites were not reached, thus making debatable the interpretation of this study and of others that used the same model.

## Effects on the stomach

### Gastric motor activity

More than 30 years ago it was observed that antipyrine *per os* (*po*) (100 mg/kg) and intravenous (*iv*) dipyrone (50, 250, and 1250 mg/kg) delay gastric emptying (GE) in rats (24,25).

Using saline as the test meal, this phenomenon was confirmed after *iv* administration of equimolar doses (240 µmol/kg) of dipyrone (80 mg/kg) and antipyrine (45.17 mg/kg), and was amplified with the inclusion of the AA metabolite (48.77 mg/kg) (26–29). Additional experiments conducted in an attempt to identify the mechanisms involved have shown that: *1*) the *iv* effect of the three drugs was dose dependent (26–29); *2*) at the dose of 240 µmol/kg a maximum effect occurred 10 min after antipyrine administration and during the first hour after the administration of dipyrone and AA (26–29); *3*) dipyrone was the only drug that caused delayed GE when applied by the intracerebroventricular (*icv*) route (26–29); *4*) electrolytic lesion of the paraventricular nucleus (PVN) of the hypothalamus abolished the *iv* effect of dipyrone (26); *5*) subdiaphragmatic vagotomy significantly reduced the effect of the three drugs (26–29); *6*) dipyrone and antipyrine administered by the parenteral route increased gastric compliance (28); *7*) *icv* administration of baclofen, a GABA_B_ receptor agonist, blocked the parenteral effect of dipyrone, AA, and antipyrine, and of dipyrone administered *icv* (27–29); *8*) apparently, there is no involvement of nitric oxide in the *iv* effect of dipyrone, opioids, or dopamine receptors (30).

Treatment of rats with capsaicin during the neonatal period (31), a procedure that causes the degeneration of non-myelinated peripheral afferent neurons (32,33), results in full blockade of the effect of the three drugs (dipyrone, AA, and antipyrine) on GE when administered *iv*, but not of the effect of *icv* dipyrone. This finding suggests that neurotoxin-sensitive afferent fibers carry the peripheral stimulus involved in GE delay. In addition, it was reported that the selective blockade of CCK_1_ or 5-HT_3_ receptors contained in these fibers, which, when activated during the digestive phase delays GE, did not modify the effect of the pyrazolone derivatives (31).

Capsaicin-sensitive afferent fibers of the common hepatic branch of the vagus project towards the rat liver (34). Since dipyrone metabolites are processed by cytochrome P450 in the rat liver (7), the effect of this drug may hypothetically stimulate these fibers through unknown mechanisms. However, results obtained with the parenteral administration of dipyrone, AA, and antipyrine to rats previously submitted to section of the hepatic branch of the vagus indicated that the fibers that pass through this branch are not involved in the mechanisms resulting in delayed GE (35).

Under non-physiological experimental conditions, there is evidence that the adrenergic system is involved in delayed GE in rats (36–40). The possibility of the participation of this system in the effect of these pyrazolone derivatives on GE was investigated and it was found that acute *iv* adrenergic blockade with guanitidine or *iv* pretreatment with propranolol (a non-selective β-adrenergic antagonist) abolished the effect of dipyrone and antipyrine on GE (41). The same procedure significantly reduced, but did not abolish, the effect of the AA metabolite. Propranolol administered *icv* did not alter the parenteral effect of the three drugs. These results suggest the participation of the adrenergic system in the delayed GE phenomenon by activation of β_1_- and/or β_2_-adrenergic receptors through the sympathetic nervous system (SNS).

Bilateral ablation of the adrenal glands, but not of the gland medulla, enhances the emptying of a liquid saline meal in rats (42). Although evidence of the participation of the SNS in the effect of the three drugs was significant, the possibility that part of the neurotransmitter might originate from the medulla of the adrenal glands could not be ruled out. However, bilateral enucleation of the adrenal medulla did not modify significantly the effect of the drugs on GE (Vinagre AM, Collares EF, unpublished results), thus supporting the hypothesis that the neurotransmitter may originate from the peripheral endings of the SNS.

β_1_- and β_2_-adrenergic receptors have been identified in the gastric mucosa and antrum muscle in rats (43), together with elevated levels of β_3_-adrenoceptor mRNA expression in the muscle fibers of the gastric fundus and the pylorus (44). A recent study using previous parenteral administration of selective β_1_-, β_2_-, or β_3-_adrenergic antagonists suggested that β_2_-adrenoceptor activation occurs in the GE delay induced by pyrazolone derivatives (45 [Bibr B46] [Bibr B47] [Bibr B48]), while the β_2_-adrenergic antagonist butoxamine completely blocked the effect of dipyrone and antipyrine on GE. In addition, this antagonist significantly reduced, but did not abolish, the effect of the AA metabolite, as observed in the previous study (41) using blockade with guanidine or pretreatment with propranolol. These results indicate that: *1*) part of the effect of dipyrone on GE is likely to be due to activation of the β_2_-adrenoceptor by the AA metabolite; *2*) a substantial component of the mechanism involved in the effect of AA (at a dose of 240 µmol/kg) is unknown.

The same study (45) also ruled out the involvement of the superior celiac-mesenteric ganglion complex of the SNS, so that the peripheral origin of the adrenergic neurotransmitter continues to be unknown. Based on available information, at least three possibilities may be suggested for the neurotransmitter origin: the splanchnic ganglia of the prevertebral ganglion chain, the sympathetic paravertebral ganglion chain for the distal part of the thorax, or the noradrenergic efferent fibers of the vagus nerve (45–49).

### Gastric mucosa

Amongst the well-known complications of the therapeutic use of NSAID, especially the non-selective COX inhibitors, are the development of gastric mucosal ulcers and changes in gastric motility (50). Considering indomethacin (a non-selective COX inhibitor) as the main model of injury to the gastric mucosa induced by NSAID in animals, a recent review on pathogenesis (50) reached the following conclusions: *1*) concomitant inhibition of COX-1 (a constitutive isoenzyme) and COX-2 (an induced isoenzyme) is needed for the development of gastric mucosa injuries; *2*) COX-1 inhibition and the consequent PGE_2_ deficiency may be responsible for gastric hypermotility which, in turn, causes vascular injury, microcirculation disorders and reduced mucosal blood flow. Gastric hypermotility may play a primary role in the development of gastric injury, occurring before later phenomena also involved in injury, such as increased neutrophil migration; *3*) prostaglandins (PGs) produced by COX-2 induction counterbalance the deleterious effect of COX-1 inhibition alone; *4*) the mechanism through which COX-1 inhibition alone induces COX-2 expression is unknown.

Contrary to the participation of gastric hypermotility in mucosal injury is the observation that non-selective COX inhibition by intragastric indomethacin delayed liquid GE in an animal model and reduced gastric tonus (51). This precedes the development of mucosal lesions and neutrophil infiltration, thus suggesting that there is no relationship between abnormal gastric motility and mucosal injury (51). Differences regarding stomach motor activity may be possibly due to the route of indomethacin administration, with the subcutaneous route having a parenteral effect (50) and the intragastric route having a local effect (51). One study favoring this interpretation showed that selective *iv* inhibition of COX-2 by valdecoxib increased gastric tonus in rats (52), suggesting that the abnormal gastric motility detected when indomethacin was administered by the parenteral route (50) may have been due to a predominant inhibition of COX-2.

Dipyrone, in contrast to the non-selective NSAIDs of the COXs, has a distinct effect on the gastric mucosa in humans and animals. Prolonged *po* treatment (14 days) of rats with dipyrone (60 mg· kg^-1^· 12 h^-1^) did not cause gastric ulcers, did not affect COX-1 expression, and did not induce COX-2 expression in the gastric mucosa (53). Orogastric administration of higher doses (1000 mg/kg) induced only few injuries to the gastric mucosa, which does not seem to relate to the significant reduction of PGE_2_ production, but is associated to inhibition of the NO/cGMP pathway and cNOS activity, which are known as important mucosal protective factors (54). *In vitro*, in contrast to the classical COX inhibitors, dipyrone and its MAA and AA metabolites apparently inhibit COX activity by either sequestering the radicals that initiate this enzyme catalytic activity or reducing the COX protein oxidative state. This indicates a distinct mechanism of action that may explain the weak anti-inflammatory effect of the drug (55).

Acute intraperitoneal (*ip*) pretreatment of rats with a single dose of dipyrone (5, 25, and 100 mg/kg) attenuated the development of ulcers induced by histamine and diethyldithiocarbamate, but not of those induced by stress. In control animals with no gastric ulcer, 5 mg/kg *ip* dipyrone increased PGE_2_ in the gastric contents (56). The protective effect of dipyrone was considered to be due to the increased synthesis and/or release of gastric mucus and to the increased gastric content of PGE_2_ (56). In addition, 3-day *ip* pretreatment with dipyrone (25, 50, and 100 mg/kg) reduced both the ulcer index and mucus secretion in rats with gastric ulcer induced by stress and diethyldithiocarbamate (57).

A significant increase in the risk of upper digestive bleeding induced by dipyrone, although twice lower than that of aspirin, has been identified in adult humans (58). Dipyrone administered *po* to healthy volunteers for two weeks (1.5 g/day) had no effect on the gastric or duodenal mucosa, with discrete damage being observed only with a higher dose (3 g/day) (59).

From another viewpoint, it is well known that, in humans, gastric-secreted acid and pepsin comprise an important factor in the genesis, maintenance, or worsening of the upper digestive tract mucosal injury, such as those found in erosive esophagitis due to gastroesophageal reflux, gastric and, especially, duodenal peptic ulcers and NSAID-induced lesions (60). No information, however, was found in the literature regarding the effect of dipyrone on the production of these two components of gastric secretion. Nevertheless, it is possible to speculate that absence of gastric ulcers associated to the use of dipyrone may be due to the interference of the drug in the regulation of gastric secretion. Thus, using an *in vivo* model for the study of gastric secretion in rats with *iv* administration of several drugs, isoprenaline (isoproterenol), a β_1_- β_2_-adrenergic agonist, inhibited the acid secretion induced by pentagastrin, an effect that was abolished by the administration of propranolol or butoxamine (61). In contrast, an *in vitro* study with an isolated stomach preparation showed that propranolol inhibited the enhancement of acid secretion induced by isoprenaline, a phenomenon that was not affected by butoxamine (61). These results lead to the conclusion that isoprenaline has an indirect (parenteral) inhibitory effect and a direct stimulatory effect on gastric acid secretion in rats, both involving activation of β-adrenoceptors (61). Since β-adrenoceptor activation by the parenteral route inhibits gastric acid secretion, it may be concluded indirectly that dipyrone, by activating these receptors in the stomach via the SNS, may have a protective effect on the gastric mucosa. However, findings from other studies did not support this interpretation. In three models of ulceration induced by ethanol, indomethacin, and stress in awake rats, *po* or *ip* pretreatment with propranolol (62) and butoxamine (63) prevented injury to the gastric mucosa, an effect attributed to reinforcement of the mucosal barrier by β-adrenergic antagonists due to the preservation of mucus-producing cells and of mucosal integrity. In anesthetized rats, using the same models on an *ex-vivo* gastric chamber preparation, butoxamine administered intragastrically or *ip* did not change the production of pepsin or acid, indicating that the antiulcerogenic action of this β_2_-adrenergic antagonist was not related to a reduced secretion of these components (63).

In conclusion, the mechanisms by which dipyrone, in contrast to nonselective NSAIDs, has a protective effect on the gastric mucosa are still unclear.

## Effect on intestinal motility

Studies using *in vitro* preparations of smooth muscle have reported controversial results regarding the spasmolytic activity of dipyrone (64–68). These conflicting results may be related to the fact that dipyrone is a pro-drug that may not have a direct effect on the muscle *in vitro* but acts indirectly through its metabolites, MAA in particular, which is generated by the drug hydrolysis (23). Ergün et al. (69) defined the following factors as being important for the *in vitro* effects of dipyrone: occurrence of non-enzymatic drug hydrolysis, local drug concentrations, and conditions related to pH, temperature and period of incubation of the muscle preparation in the organ bath. All these conditions must be strictly controlled in order to obtain reliable results.

Little information is available about the action of dipyrone on intestinal motility. Small bowel peristalsis of guinea pig intestine is not blocked when exposed *in vitro* to 10–100 µM dipyrone in a bath for 60 min (70). In contrast, at higher concentrations (100–500 µM), the drug had a myogenic spasmolytic effect for 30 min in the ileum (71). On the other hand, dipyrone (50 and 250 mg/kg) administered *in vivo* to rats did not change the intestinal transit (25), and the drug had no effect on experimentally induced contractions of the cecum of ponies (72), and on the jejunal or colonic (pelvic flexure) motility of these animals (73). In the awake opossum, without the addition of any other active principle, the drug was not able to break the migrating motor complex (MMC) cycle (74 [Bibr B75]).

Whether the drug has or not a spasmolytic action on intestinal colic is inconclusive, since experimental studies on animals have suggested that the positive clinical results observed in humans and animals may be due to its analgesic property.

## Effect on the biliary tract

Sphincter of Oddi dysfunction may manifest clinically by recurrent abdominal pain of the "biliary type" and/or episodes of acute pancreatitis, with abnormal motility of the sphincter and a significant increase in its basal pressure having been identified (75). In this condition, dipyrone was proven more effective in controlling acute biliary colic pain than opioids or pure spasmolytic drugs (76). However, a systematic review and meta-analysis found that NSAIDs were more effective in both control of biliary colic and prevention of potential progression to cholelithiasis than opioids, spasmolytic drugs, or dipyrone, probably because of the diversity of their pharmacological actions, locally and in the CNS (77). A more recent meta-analysis that did not include dipyrone also indicated that NSAIDs were superior to placebo and spasmolytic drugs, but not to opioids, in the control of biliary colic, additionally indicating a reduction in the frequency of complications related to the biliary tract (78).

Dipyrone was found to reduce the tonus of the sphincter of Oddi in humans, which possibly indicates another mechanism of action on biliary colic, in addition to the analgesic effect (79). Conversely, dipyrone did not change the biliary pressure or the sphincter of Oddi myoelectric activity in the non-sedated opossum (74). No information was found in the literature about the effect of the drug on the biliary pathways in rats.

The sphincter of Oddi in species such as the opossum, the guinea pig, and the rabbit has different anatomical and functional motor characteristics than those of humans (80). This is not the case for carnivore animals (dogs and cats), in which the structure and function of the sphincter of Oddi were found to be similar to those of humans (80,81).

In dogs and cats, the sphincter of Oddi is under direct adrenergic control from the sympathetic plexus and ganglia, with the participation of adrenergic axons of the vagus being apparently questionable (82). In addition, sympathetic stimulation causes relaxation of the gallbladder through a direct effect, which was found in dogs and cats and in humans as well (83).

Adrenergic fibers were found to partially mediate relaxation of the isolated gallbladder of dogs, with a significant component of this effect depending on non-cholinergic, non-adrenergic neurotransmitters (nitric oxide, intestinal vasoactive peptide, and carbon monoxide) (84).

In an isolated preparation of the sphincter of Oddi of cats, it was demonstrated that α-adrenoceptor activation promotes contraction, whereas activation of β-adrenoceptors promotes relaxation (85). In anesthetized cats, the sphincter of Oddi tonus increased *in vivo* due to the activation of α-adrenoceptors and decreased with the activation of β-adrenoceptors probably with the involvement of the β_2_-adrenergic subtype (86). In addition, in anesthetized cats, the activation of the β_2_-adrenoceptor induced relaxation of the sphincter of Oddi, a phenomenon antagonized by propranolol and by the selective blockade of the β_2_-adrenergic receptor (87).

Although the use of results from experiments in animals to interpret what occurs in humans is limited, a reasonable amount of information allows the speculation that the activation of β_2_-adrenoceptors by metabolites of dipyrone (the same involved in delaying GE in rats) may contribute, at least in part, to the drug spasmolytic effect on biliary colic. In this clinical situation, this effect may occur in addition to the analgesic property of the drug. However, the demonstration of the participation of the β_2_-adrenergic subtype in the effect of dipyrone in humans may be questioned, taking into account that the selective adrenergic antagonists currently available also block β-adrenoceptors in the respiratory apparatus, provoking bronchospasm (88).

## Relationship between the antinociceptive and antispasmodic effects of dipyrone

A few studies have examined the antinociceptive effect of dipyrone in rats (20,21). Hernández-Delgadillo et al. (89) and Silva-Moreno et al. (90) evaluated this effect using extremely high doses of *iv* dipyrone, i.e., 7.5 and 5 times higher, respectively, than those employed in studies of the effect of the drug on GE of liquids in rats (26–31,35,41,45). Rupp et al. (25), in a study of the effect of dipyrone on GE in rats, used 1250 mg/kg *iv* as the maximum dose. However, the authors detected a significant delay of GE with a lower dose (50 mg/kg). In our first observation (26), the delay of GE was already significant with a dose of 40 mg/kg. In subsequent studies, the dose of 80 mg/kg was used as reference. The relationships between the effects of dipyrone on GE of rats and its antinociceptive influence is therefore unknown, and whether this is dependent on dosage is uncertain. On the basis of the dose-response curves of the two mentioned studies (89,90), we observed that the dose of 80 mg/kg is lower than the lower limit of the curve of the antinociceptive effect (percent of maximum possible effect) of this drug in rats. Although this is a rough interpretation, it may indicate that there is no relationship between the two effects.

## Unknown effects on the digestive tract

We did not find information in the literature available on the effect of dipyrone on the motor activity of the stomach in humans. In addition, there is no information on the effect of the drug on the motor activity of other parts of the gastrointestinal tract, such as the esophagus and the lower esophageal sphincter, the pylorus, or the internal anal sphincter, both in humans or animals.

## Research proposals

The available information regarding the effect of pyrazolone derivatives on GE in rats is limited, which indicates the need of further studies in order to clarify several points that might help understand the spasmolytic effect of dipyrone on the digestive tract. Among them, the following points should be clarified: *1*) degree of participation of the MAA and AA metabolites and others in the effect of dipyrone; *2*) mechanism(s) by which pyrazolone derivatives activate the capsaicin-sensitive afferent pathways; *3*) mechanism(s) additional to the activation of the β2-adrenoceptor mediating the effect of the AA metabolite delaying GE; *4*) origin of the norepinephrine mediator in the SNS; *5*) participation of the PVN in the integration between the spinal and/or vagal afferent pathways; *6*) the efferent pathways of the vagus (adrenergic) and the SNS involved in the various effects of dipyrone.
